# CDK1-driven phosphorylation networks promote glioblastoma progression via MAP1B-mediated microtubule destabilization

**DOI:** 10.3389/fonc.2025.1646698

**Published:** 2026-01-14

**Authors:** Jun-Tao Li, Meng-Da Li, Yong-Ji Guo, Zhi-Xiao Li, Rong-Jun Qian, Chun-Xiao Ma

**Affiliations:** 1 Department of Neurosurgery, Henan Provincial People’s Hospital, Zhengzhou, China; 2 Department of Neurosurgery, People’s Hospital of Henan University, Zhengzhou, China; 3 Department of Neurosurgery, People’s Hospital of Zhengzhou University, Zhengzhou, China

**Keywords:** Cdk1, GBM, MAP1B, microtubule, phosphorylation

## Abstract

**Background:**

Glioblastoma (GBM) is the most aggressive and prevalent malignant brain tumor in adults, with poor prognosis despite current therapies. Cyclin-dependent kinase 1 (CDK1), a master regulator of cell cycle progression, has been implicated in oncogenesis, but its downstream phosphorylation network in GBM remains incompletely defined.

**Methods:**

CDK1 expression was examined in clinical GBM tissues and cell lines. Functional studies were performed in U251 cells using CDK1-specific shRNAs. Label-free phosphoproteomic profiling and bioinformatics analyses were conducted to map CDK1-regulated signaling pathways and substrates. Prognostic associations were evaluated using Clinical Proteomic Tumor Analysis Consortium (CPTAC) datasets, and functional assays were used to validate candidate substrates.

**Results:**

CDK1 was significantly upregulated in GBM tissues, and its knockdown suppressed proliferation, migration, and invasion of U251 cells. Phosphoproteomic analysis identified 15,156 phosphorylation sites, of which 2,836 were significantly altered by CDK1 inhibition, implicating pathways related to cell cycle regulation, DNA replication, and DNA damage repair. Subcellular localization revealed nuclear enrichment, including phosphorylation changes in RB1 and TP53. Importantly, CDK1-mediated hyperphosphorylation of microtubule-associated protein 1B (MAP1B) at multiple residues (Ser832, Ser1260, Ser1899, Ser1939, Ser2209, Ser2271) correlated with poor prognosis and promoted microtubule destabilization. Functional assays confirmed that MAP1B knockdown impaired GBM cell growth, migration, and invasion.

**Conclusion:**

This study demonstrates that CDK1 is a critical oncogenic driver in GBM, regulating broad phosphosignaling networks and promoting tumor progression via MAP1B-dependent microtubule destabilization. MAP1B phosphorylation emerges as a potential prognostic biomarker. These findings support the development of CDK1-targeted therapies, alone or combined with microtubule-stabilizing agents, for improved GBM management.

## 1 Introduction

Glioblastoma (GBM) is the most common primary malignant brain tumor in adults, accounting for 48.3% of malignant central nervous system (CNS) tumors. GBM is characterized by uncontrolled proliferation, invasion, and strong resistance to therapy. Current treatment options—surgical resection, radiotherapy, and chemotherapy—yield a median survival of only 14.6 months (1, 2). Despite decades of research and multimodal treatment strategies, significant improvements in survival outcomes remain limited (3, 4).

Phosphorylation is a fundamental post-translational modification involved in diverse biological processes and is critical in tumor cell regulation. Protein kinases, the “writers” of phosphorylation, have attracted significant interest in cancer drug discovery (5, 6). Mutations in kinases alter downstream signaling pathways and promote tumorigenesis, while kinase inhibitors can suppress tumor progression by blocking kinase activity.

Cyclin-dependent kinases (CDKs), a family of serine/threonine kinases, regulate the cell cycle through substrate phosphorylation and CDK–cyclin complex formation (7). CDK1, in particular, regulates the G2–M transition and promotes mitotic entry via cyclin B binding. CDK1 activity and expression strongly influence proliferation and division, making it a promising therapeutic target. Given its frequent overexpression in GBM, delineating CDK1’s downstream phosphorylation network is essential to understanding its oncogenic role (7–12).

In this study, we demonstrate that CDK1 is highly expressed in GBM. Knockdown of CDK1 in the U251 cell line suppressed cell proliferation. Using label-free phosphoproteomics, we mapped global phosphorylation changes following CDK1 inhibition and identified key downstream substrates and pathways. Our analysis revealed that CDK1 regulates phosphorylation of crucial transcription factors (e.g., RB1, TP53) and oncogenic drivers, reinforcing its role in GBM pathogenesis. Importantly, we identified MAP1B as a novel CDK1-regulated substrate whose hyperphosphorylation correlates with poor prognosis and microtubule destabilization.

## 2 Materials and methods

This research was approved by the Ethics Committee of Henan Provincial People’s Hospital (Zhengzhou University People’s Hospital) and conducted in accordance with the Declaration of Helsinki. Written informed consent was obtained from patients prior to surgery.

### 2.1 Antibodies

CDK1 Polyclonal antibody (19532-1-AP, proteintech) was used for immunohistochemistry(IHC), western blot and immunofluorescence(IF). MAP1B antibody(EPR27225-25) was used for immunoprecipitation(IP). Phospho-(Ser/Thr) Phe Antibody (#9631, Cell Signaling Technology), MAP1B Polyclonal antibody(21633-1-AP, proteintech) and β-actin Polyclonal antibody(20536-1-AP, proteintech) were used for western blot. Acetyl-α-Tubulin antibody(#5335, Cell Signaling Technology) was used for immunofluorescence. Goat Anti-Rabbit IgG HRP(SA00001-2, proteintech) were used for immunohistochemistry and western blot. Goat anti-Rabbit IgG (H+L) Cross-Adsorbed ReadyProbes™ Secondary Antibody, Alexa Fluor™ 488 were used for immunofluorescence.

### 2.2 Tissue collection

Tumor and adjacent tissues were obtained from five patients who underwent GBM resection between January 2021 and December 2024 at Henan Provincial People’s Hospital.

### 2.3 Cell line and shRNA

Human GBM cell line U251 was obtained from the American Type Culture Collection (ATCC). CDK1and MAP1B specific shRNAs were obtained from a commercial RNA interference library. The sequences of shCDK1–1 and shCDK1–2 were TCGAAAATGTTAATCTATG and AGGTTATATCTTTG, and the sequence of shMAP1B was GATACTCTATCCGATGTTG.

### 2.4 Western blot

Cells were lysed in NP-40 buffer containing protease inhibitors (Roche). Lysates were centrifuged (12,000 g, 15 min, 4°C), and protein concentration was measured using a micro-BCA kit (Thermo Fisher). Samples were separated by SDS-PAGE, transferred onto nitrocellulose membranes, and blocked in 5% non-fat milk. Membranes were probed with primary antibodies overnight at 4°C, followed by HRP-conjugated secondary antibodies at room temperature for 1 h. Signals were visualized using ECL reagents (Pierce), and band intensities were quantified with ImageJ software.

### 2.5 Immunohistochemistry

Tissues were formalin-fixed, paraffin-embedded, and sectioned (4–6 µm). Following deparaffinization and rehydration, antigen retrieval was performed (citrate buffer, pH 6.0 or EDTA, pH 9.0). Endogenous peroxidase was blocked with 3% H_2_O_2_, and nonspecific binding was blocked with 5% normal serum. Slides were incubated with primary antibodies overnight at 4°C. After washing, HRP-conjugated secondary antibodies were applied, and DAB was used for chromogenic detection. Nuclei were counterstained with hematoxylin. Negative and positive controls were included to ensure specificity.

### 2.6 Immunofluorescence staining

Cells grown on coverslips were fixed with 4% paraformaldehyde or cold methanol. After permeabilization with 0.1–0.5% Triton X-100, nonspecific binding was blocked with 5% BSA or 10% normal serum. Primary antibodies were incubated overnight at 4°C, followed by fluorophore-conjugated secondary antibodies (Alexa Fluor 488). Nuclei were counterstained with DAPI. Images were captured with a confocal microscope.

### 2.7 Protein extraction and digestion for phosphoprotome

Cells were lysed in 8 M urea containing protease and phosphatase inhibitors. Lysates were sonicated and centrifuged (12,000 g, 10 min, 4°C). Proteins were quantified by BCA assay, reduced with DTT, alkylated with iodoacetamide, digested with trypsin (1:50), and desalted using Strata X SPE columns.

### 2.8 Phosphopeptide enrichment

First, the peptide mixture was incubated with the Immobilized Metal-Affinity Chromatography (IMAC) microsphere suspension in loading buffer consisting of 50% acetonitrile and 0.5% acetic acid. To remove the non-specifically adsorbed peptides, the IMAC microspheres were sequentially washed with 50% acetonitrile and 0.5% acetic acid, followed by 30% acetonitrile and 0.1% trifluoroacetic acid. The enriched phosphopeptides were then eluted with 10% NH_4_OH through vibration, and subsequently collected and lyophilized for mass spectrometer analysis.

### 2.9 Mass spectrometer

Peptides were analyzed using EASY-nLC 1200 coupled to an Orbitrap Exploris 480. Separation was achieved on a 25 cm reversed-phase column with a 90-min gradient. The Orbitrap was operated at 60,000 resolution for MS1 and 30,000 for MS/MS.

### 2.10 Database search

The resulting MS/MS data were processed using the Proteome Discoverer search engine (v.2.4) to search tandem mass spectra against the Homo_sapiens_9606_SP_20230103.fasta (20389 entries) database, concatenated with a reverse decoy and contaminants database. Trypsin (full) was specified as the cleavage enzyme, allowing up to 2 missed cleavages. The minimum peptide length was set at 6, and the maximum number of modifications per peptide was set at 3. The mass error was set to 10 ppm for precursor ions and 0.02 Da for fragment ions. Carbamidomethylation on cysteine was specified as a fixed modification, while oxidation on methionine, acetylation on protein N-terminal, loss of methionine, loss of methionine with acetylation, and phosphorylation on serine or threonine were specified as variable modifications. The false discovery rate (FDR) for proteins, peptides, and PSMs was adjusted to less than 1%.

### 2.11 Phosphoproteome bioinformatics data analysis

The phosphoproteome intensities were normalized by subtracting the median intensities of various samples. All the indentified phosphoproteins were annotated for categories in the R software, including GO’s biological process (BP), molecular function (MF), cellular component (CC), subcellular localization, is TF and KEGG pathways. The differentially expressed phosphosites were analyzed for enrichment in GO and KEGG pathways. Heatmaps were generated to visualize the (z-score normalized) expression levels of related phosphosites.

### 2.12 Prognostic analysis of CPTAC phosphroteome

Prognostic phosphosites were identified from CPTAC phosphoproteomics data by first preprocessing raw datasets (retaining phosphosites quantified in ≥70% samples with kNN imputation and log2 normalization), followed by univariate Cox regression analysis (FDR<0.05) adjusted for clinical covariates. Significant phosphosites were stratified by median abundance for Kaplan-Meier survival analysis (log-rank test).

## 3 Results

### 3.1 CDK1 is upregulated in GBM and promote GBM growth

**Figure 1:**
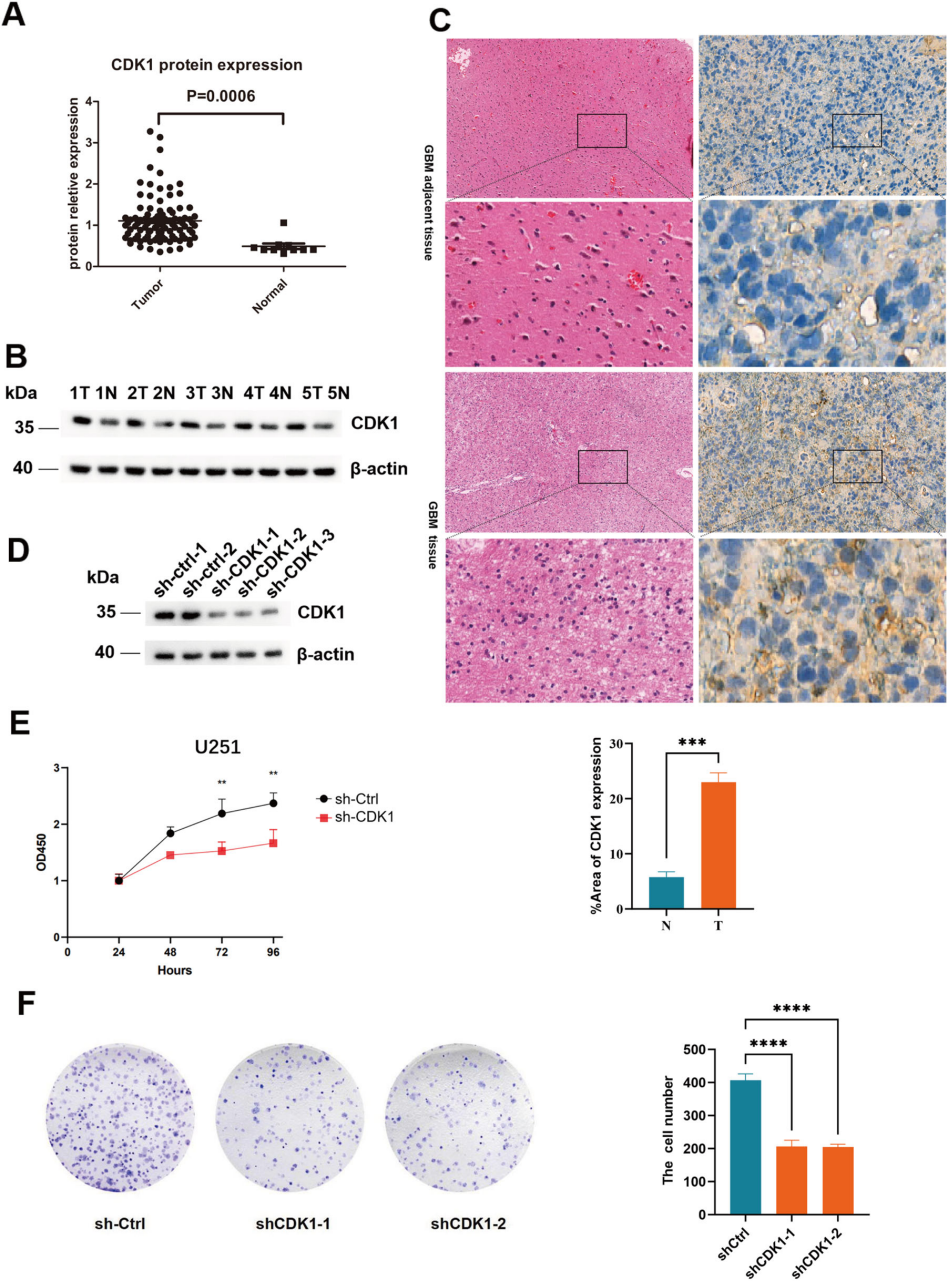
CDK1 is upregulated in GBM and promotes tumor proliferation in U251 cells. **(A)** Bioinformatics analysis of CDK1 expression in GBM Data from CPTAC database show significantly elevated CDK1 protein levels in tumor tissues (n=99) compared to normal brain tissues (n=10). **(B)** Validation of CDK1 protein expression by western blot. Representative blots of CDK1 in GBM tumor tissues (T) and paired tumor adjacent normal tissues (N) from 5 patients, β-actin served as loading control. **(C)** HE staining and CDK1 IHC staining results in GBM tumor tissue and adjacent non-tumor tissue, ***p < 0.001 (t-test). **(D)** Efficient CDK1 knockdown in U251 cells. Western blot analysis of shCDK1 and shCtrl cells. **(E)** Cell growth curve of shCDK1 and shCtrl U251 cells, ** P < 0.01(t-test). **(F)** Colony formation result of shCDK1 and shCtrl U251 cells, ***p < 0.001 (t-test).

To investigate the expression status of CDK1 in GBM, we analyzed the proteomics data from Clinical Proteomic Tumor Analysis Consortium (CPTAC), which revealed significant overexpression of CDK1 in tumor tissues compared to normal adjacent tissues(**Figure 1A**) (13). Western blot and IHC confirmed high CDK1 expression in tumors (**Figures 1B, C**). To investigate the functional role of CDK1 in GBM, we constructed a lentiviral vector expressing shRNA specifically targeting CDK1 and infected U251 cells with the vector. Western blot analysis was used to validate the knockdown efficiency (**Figure 1D**). We found that the growth rate of U251 cells infected with the CDK1 shRNA vector was significantly slower compared to sh-control(shCtrl) cells (**Figures 1E, F**). This indicates that CDK1 is a crucial protein for the growth of U251 cells.

### 3.2 Global profiling of the phosphoproteome in CDK1-knockdown U251 cells

**Figure 2:**
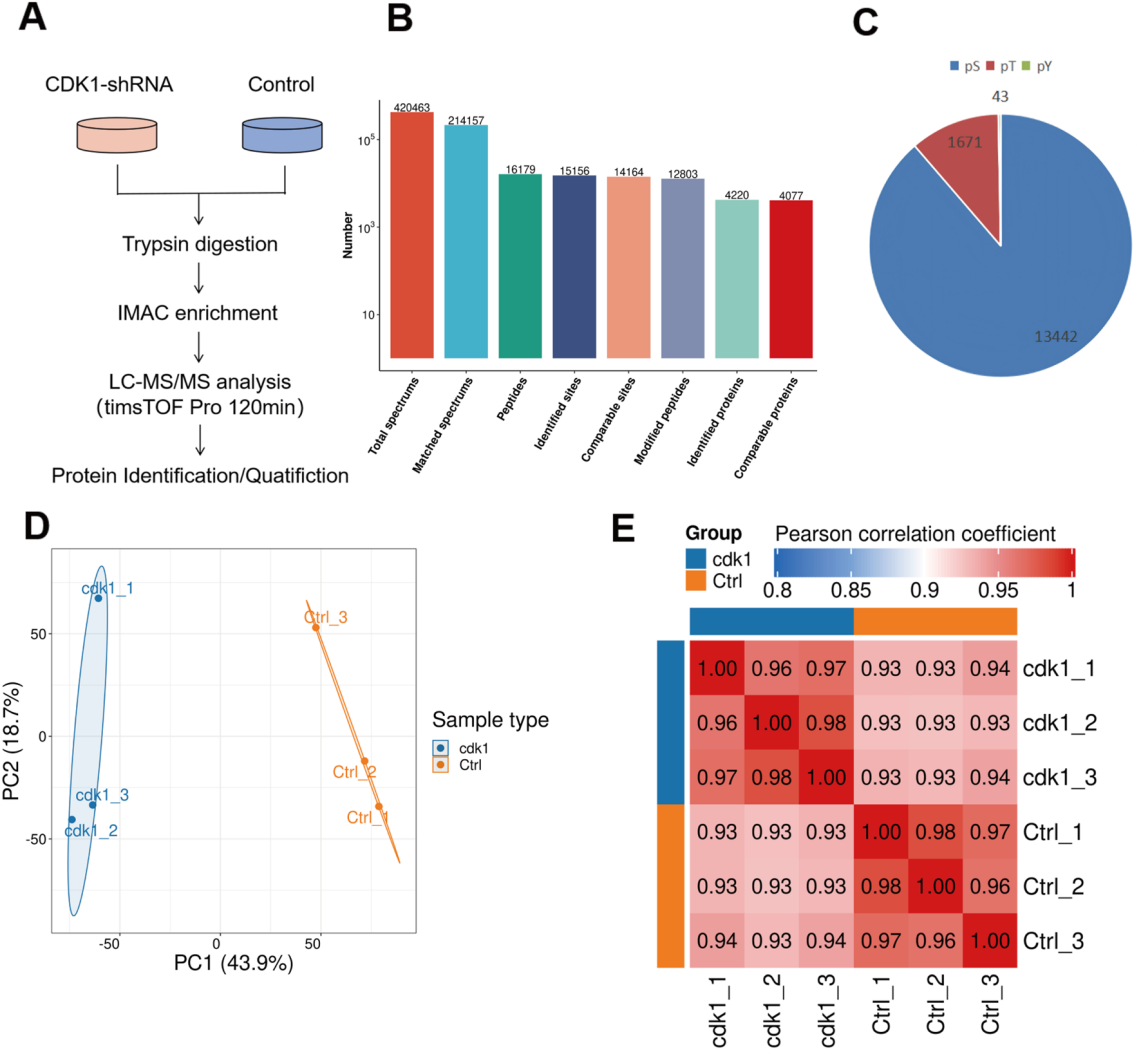
Summary of the phosphoproteomic results from shCDK1and shCtrl cells **(A)** The systematic workflow for quantitative profiling of global phosphoproteome. Label free quantification were used in this study. **(B)** Number of total spectrums, matched spectrums, peptides, identified peptides, comparable sites, modified sites, identified proteins and comparable proteins in the study. **(C)** Distribution of phosphorylated amino acids. Pie chart represents the relative abundance of phosphoserine (blue), phosphothreonine (red), phosphotyrosine (green). **(D)** Principal component analysis (PCA) of the phosphoproteome. Sample clustering demonstrates clear separation between CDK1 (blue) and shCtrl (orange) conditions. **(E)** Correlation matrix of the phosphoproteomic data. The intensity of the color corresponds to correlation strength.

To further explore the function of CDK1 in GBM from the perspective of phosphorylation modification, U251 cells stably expressing CDK1-specific shRNA (shCDK1) and control cells were collected to performed label-free phosphoproteomic analysis. The workflow is shown in **Figure 2A**. In our study, a total of 12,803 phosphorylated peptides and 15,156 phosphorylation sites on 4,220 proteins were identified (**Figure 2B**). The 15,156 phosphosites included 13,442 phosphoserine sites (88.69%), 1,671 phosphothreonine sites (11.03%), and 43 phosphotyrosine sites (0.28%). The distribution is summarized in **Figure 2C**.

To obtain a quantitative signature of the phosphoproteomic data, Principal Component Analysis (PCA) and Pearson correlation parameters were performed (**Figure 2D**). The shCDK1 group and shCtrl group were clearly discriminated in the PCA result. The correlation analysis showed that the intra-group Pearson correlation coefficients for the shCDK1 and shCtrl groups were significantly higher than the inter-group coefficients (**Figure 2E**). This shows that our samples have good repeatability.

**Figure 3:**
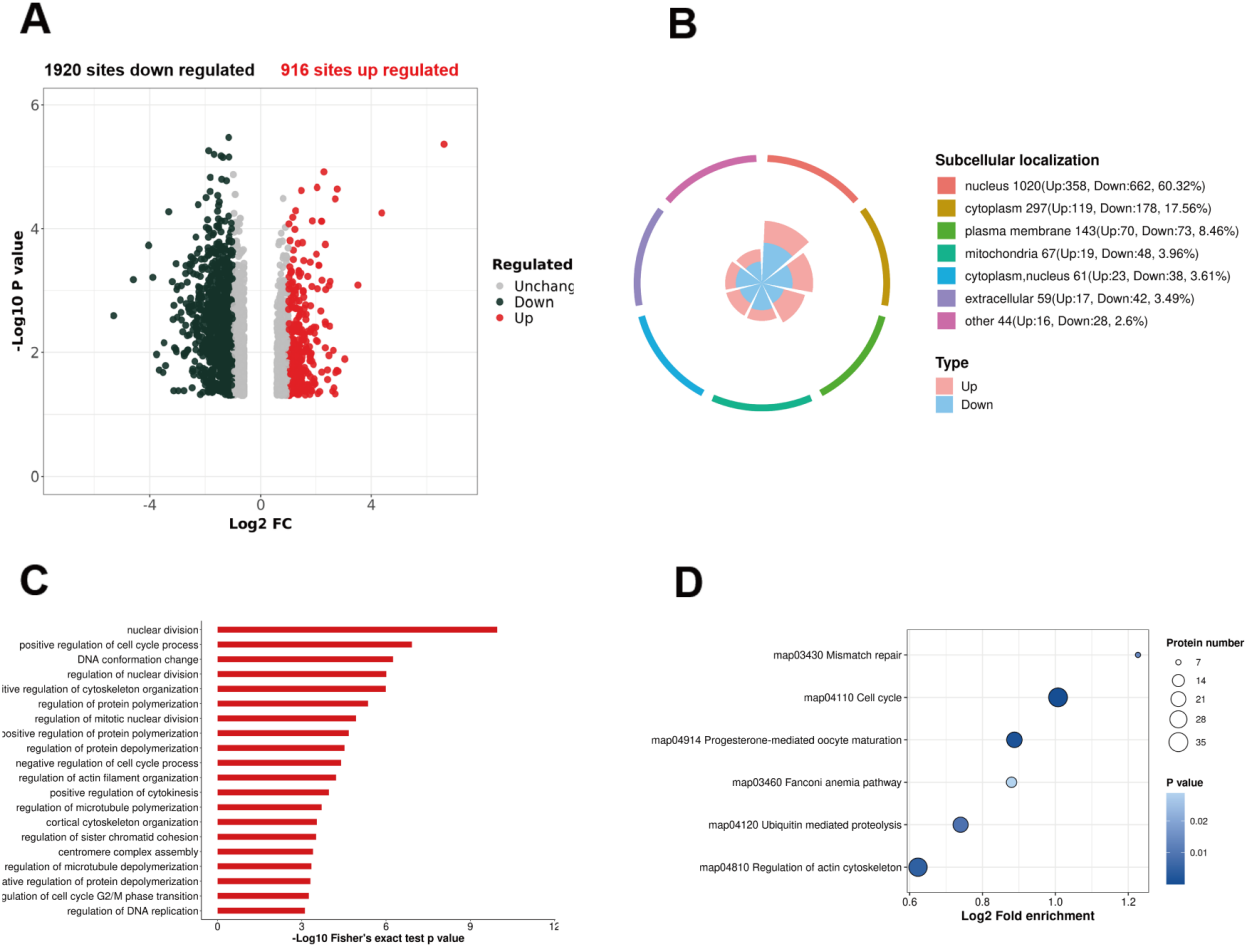
Comprehensive analysis of CDK1-regulated phosphoproteome. **(A)** Volcano plot showing differentially expressed phosphosites. Red represents upregulated phosphosites, black represents downregulated phosphosites. **(B)** Subcellular localization analysis of CDK1-regulated phosphoproteins. **(C)** GO biological process enrichment analysis of CDK1-regulated phosphoproteins. **(D)** KEGG pathway analysis of CDK1-regulated phosphoproteins. Bubble size represents the number of genes in the pathway. Bubble color indicates the significance of enrichment (−log_10_(p-value)), Deeper blue is more significant.

By comparing shCDK1 and shCtrl cells, a total of 1920 phosphosites on 1069 proteins that were significantly decreased in shCDK1 cells, and 916 phosphosites on 622 proteins that were significantly increased in shCDK1 cells were identified based on a 1.5-fold difference and a significance level of P < 0.05 in a Welch t-test (**Figure 3A**). Then Subcellular localization, Gene Ontology (GO) categorization, KEGG pathway and transcription factor annotation analysis were performed with the differenttly expressed phosphorylation sites.

### 3.3 Subcellular localization analysis reveals CDK1 regulation of nuclear transcription factors

Subcellular localization analysis revealed that differentially expressed phosphosites were predominantly localized to in the nucleus, cell membrane, and cytoplasm (**Figure 3B**). Since CDK1 is mainly localized in the nucleus, this is consistent with our expectations for the results. Given that the activation of transcription factors depends on phosphorylation modification, phosphorylated proteins regulated by CDK1 have been annotated in the is_TF database, revealing a total of 242 phosphorylation sites in 139 transcription factors were regulated by CDK1 (**Supplementary Table S1a**). Retinoblastoma 1(RB1) protein, a crucial regulator of cell cycle progression, plays significant roles in cellular senescence, differentiation, apoptosis, and tumorigenesis. It regulates the cell cycle by interacting with E2F, the phosphorylation of RB1 is critical for E2F activity and is regulated by CDK4/6. In this study, we found that RB1 phosphorylation was significantly reduced in the shCDK1 group. TP53, which functions as a tumor suppressor in numerous tumor types, demonstrated decreased expression at ser 6 and ser315 in our study (14). This is consistent with previous reports as it serves as a classic substrate for CDK1 (15, 16). The phosphorylation modifications of numerous transcription factors are regulated, indicating that CDK1 plays a crucial role in GBM transcriptional regulation.

### 3.4 Pathway enrichment analysis implicates CDK1 in cell cycle and DNA integrity processes

To further elucidate the protein functions modulated by CDK1-regulated phosphorylation, we performed comprehensive enrichment analyses using GO, KEGG, and Wiki-Pathways databases. The results from the biological process enrichment analysis revealed that the majority of downregulated pathways are intimately associated with nuclear division, positive regulation of the cell cycle process, and DNA conformation changes. Notably, biological processes pertaining to cell cycle progression and DNA damage repair were also significantly enriched (**Figure 3C**). The KEGG enrichment analysis further highlighted that the downregulated pathways are primarily related to the cell cycle, progesterone-mediated oocyte maturation, and regulation of the actin cytoskeleton (**Figure 3D**). These findings underscore the profound impact of CDK1 knockdown on crucial cellular processes, including cell cycle regulation, DNA replication, and DNA damage repair. The TP53/cell cycle signaling pathway is indeed a crucial oncogene pathway in GBM, and the regulation of TP53 and the cell cycle by CDK1 highlights its potential as a therapeutic target for GBM treatment.

### 3.5 Identification of CDK1 candidate substrates in U251 cells

**Figure 4:**
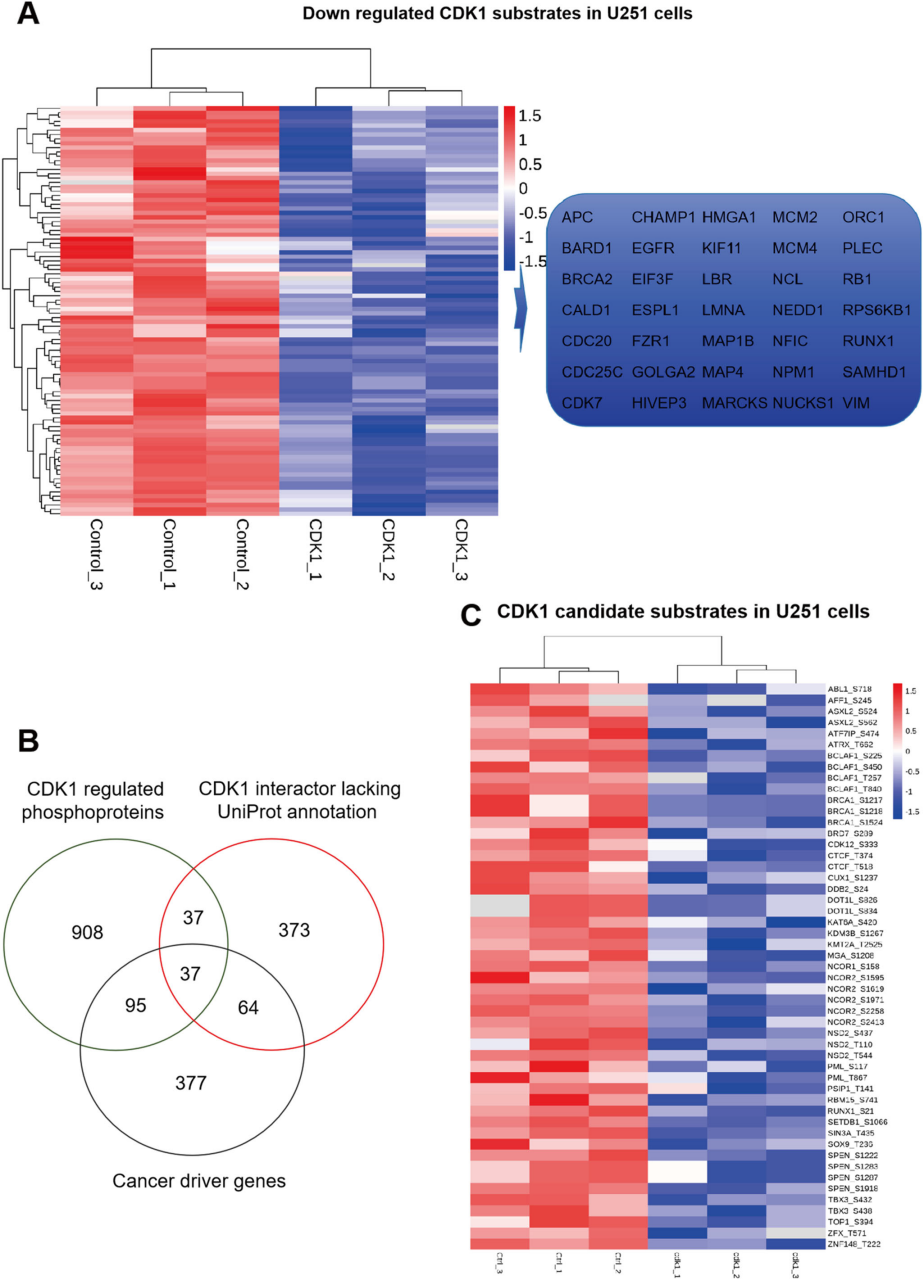
CDK1 substrates and candidate substrates in GBM cells. **(A)** Heatmap of 35 downregulated CDK1 substrates in U251 cells. Red indicates upregulated values relative to the mean, while blue indicates downregulated values relative to the mean. The intensity of the color corresponds to the magnitude of the deviation. **(B)** Overlap analysis of CDK1-regulated phosphoproteins (green), CDK1 BioGRID interactor lacking uniprot annotation (red), and cancer driver genes (black). **(C)** Heatmap of 37 candidate CDK1 substrates in U251 cells. Red indicates upregulated values relative to the mean, while blue indicates downregulated values relative to the mean. The intensity of the color corresponds to the magnitude of the deviation.

As a classic kinase, CDK1 has many substrates that have been reported. According to UniProt, 87 proteins have been reported to undergo phosphorylation by CDK1 (17–34). In our current study, we successfully identified 55 of these proteins, constituting a significant proportion of the known CDK1-regulated phosphoproteome. Notably, 35 of these identified proteins exhibited a downregulated phosphorylated state. As is shown in **Figure 4A**. This suggests that CDK1 knockdown in U251 cell lines leads to significant alterations in the phosphorylation levels of substrate proteins, and these substrate changes can be well detected using phosphoproteome.

Proteins interacting with CDK1 frequently undergo phosphorylation modifications, a key regulatory mechanism in cellular processes. To systematically characterize CDK1’s substrate spectrum, we triangulated three datasets: (1) CDK1 physical interactors from BioGRID lacking Uniprot annotation (n=491), (2) CDK1-dependent phosphoproteins from our phosphoproteomics, and (3) oncogenic drivers (35). This revealed 109 CDK1-regulated phosphosubstrates, 37 of which were annotated cancer drivers(**Figures 4B, C**; **Supplementary Table S1b**). These cancer driving genes may be candidate substrates of CDK1, indicating that CDK1 plays an important role in tumor development.

### 3.6 Phosphorylated MAP1B is a potential prognostic biomarkers in GBM regulated by CDK1

**Figure 5:**
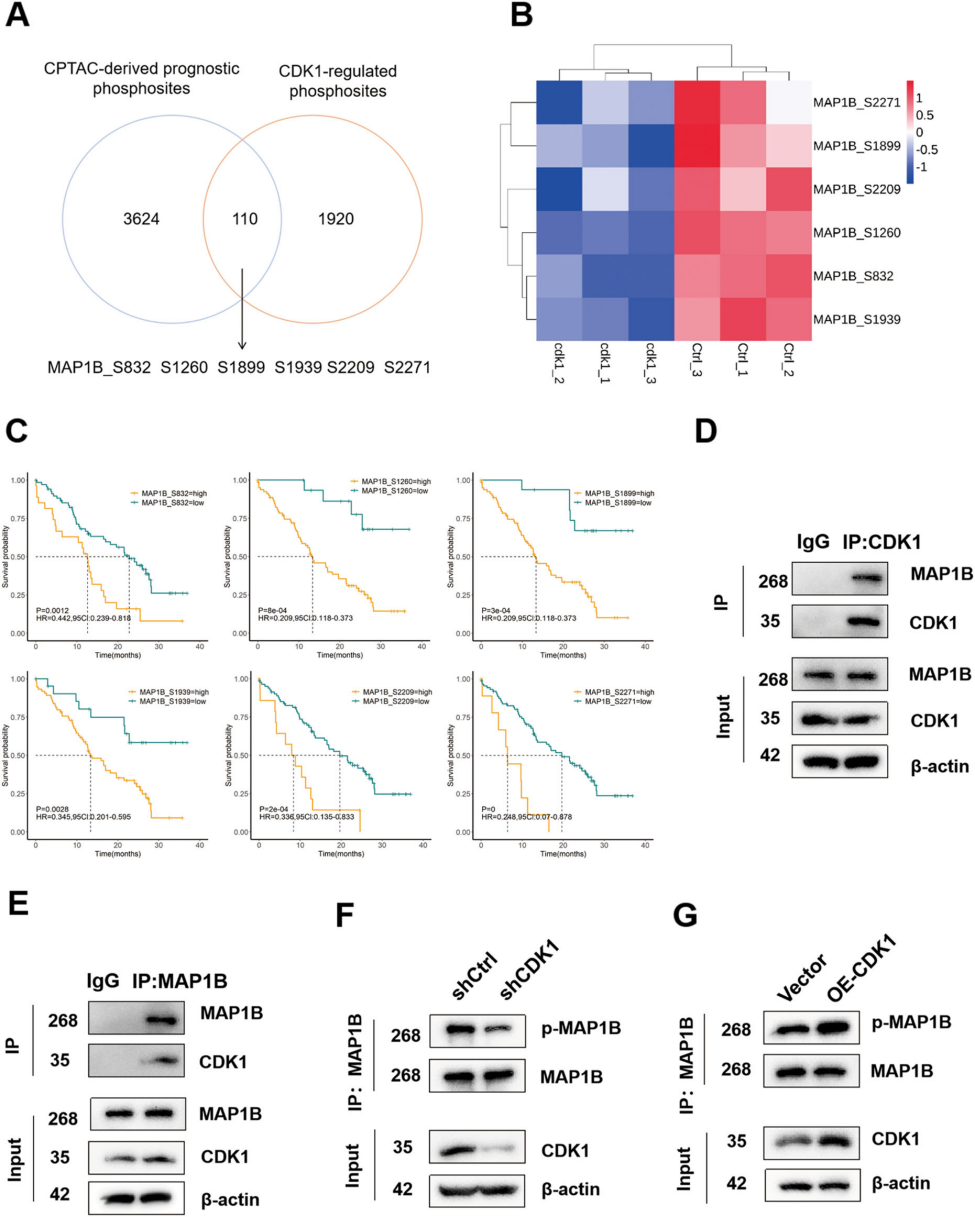
CDK1-mediated MAP1B phosphorylation is positively correlated with the prognosis of GBM. **(A)** Overlap analysis of CPTAC phosphoproteome data (blue) with CDK1-regulated phosphosites (red). **(B)** Heatmap of MAP1B phosphosites in the shCDK1 and shCtrl groups. Red indicates upregulated values relative to the mean, while blue indicates downregulated values relative to the mean. The intensity of the color corresponds to the magnitude of the deviation. **(C)** Prognostic significance of MAP1B phosphorylation sites. Kaplan-Meier survival analysis of GBM patients stratified by phosphorylation levels at S832, S1260, S1899, S1939, S2209, S2271(log-rank test, p < 0.05). High phosphorylation correlates with poor overall survival (n=99). **(D, E)** CoIP showing interactions between CDK1 and MAP1B. **(F)** Detecting pS/T phosphorylation of MAP1B in shCDK1 and shCtrl U251 cells. **(G)** Detecting pS/T phosphorylation of MAP1B in overexpressed-CDK1(OE-CDK1) and empty vector(Vector) U251 cells.

The clinical detection of GBM by the CPTAC encompasses proteomics and phosphorylation profiling, upon which we conducted a thorough prognostic analysis. Our findings revealed that 70 phosphosites on 61 proteins were significantly associated with prognosis, and were downregulated in the shCDK1 group compared to the shCtrl (**Figure 5A**). Among these, the microtubule-associated protein MAP1B stood out, with most phosphorylation modification sites linked to prognosis and regulated by CDK1(**Figure 5B**). Hyperphosphorylation of MAP1B at Ser832, Ser1260, Ser1899, Ser1939, S2209, and S2271 was associated with poorer patient prognosis (**Figure 5C**). The CO-IP experiment was performed to demonstrate the interaction between CDK1 and MAP1B(**Figures 5D, E**). Inhibition of CDK1 significantly reduced the serine and threonine (pS/T) phosphorylation levels of MAP1B, while overexpression of CDK1 increased the pS/T phosphorylation levels of MAP1B (**Figures 5F, G**). This indicates MAP1B is a substrate of CDK1.

**Figure 6:**
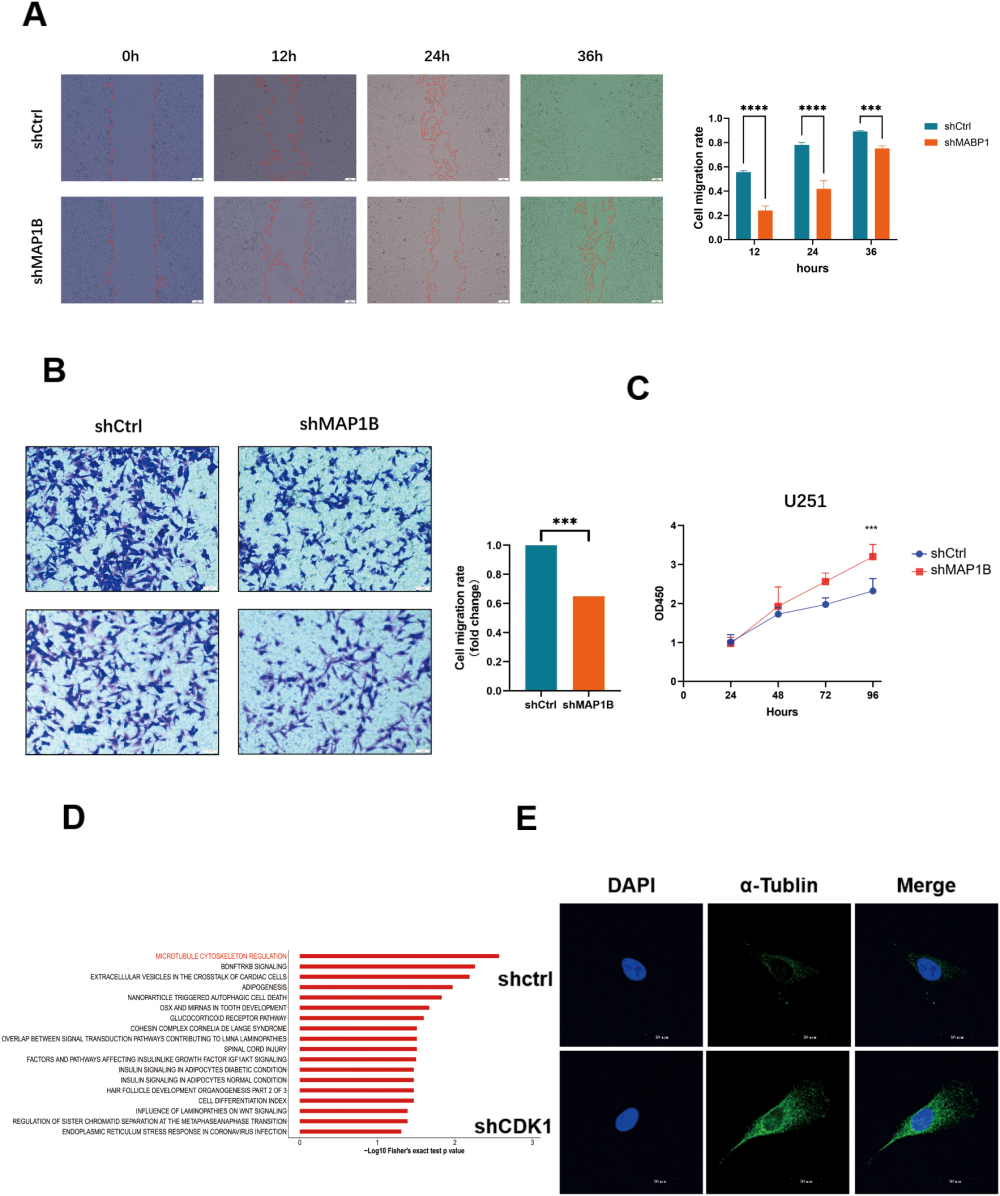
CDK1-mediated phosphorylation of MAP1B regulates microtubule stability in U251 cells. **(A)** Wound healing assays showing delayed gap closure in shMAP1B cells compared to shCtrl cells over 36 hours, ***p < 0.001 (t-test), ****p < 0.0001 (t-test). **(B)** Transwell migration assays confirmed the impaired migration ability of shMAP1B cells. ***p < 0.001 (t-test). **(C)** CCK-8 proliferation assays revealed a significant decrease in cell viability in shMAP1B cells ***p < 0.001 (t-test). **(D)** Wiki-pathways analysis of differently expressed phosphoproteins. **(E)** Representative photographs of alpha tubulin staining of in U251 cells. Scale bar: 20 μm. Cells were stained with an antibody against acetyl-α-tubulin (green). Nuclei were counterstained with DAPI (blue). Scale bar, 20 μm.

MAP1B is well-known for facilitating the tyrosination of alpha-tubulin in neuronal microtubules. In several cancer types, MAP1B has been implicated in tumor progression, where it facilitates proliferation, metastasis, and invasion. To assess the functional role of MAP1B, we performed functional assays in U251 cells, including the CCK-8 assay for proliferation, wound healing assay for migration, and Transwell assay for invasion. The results collectively demonstrated that knockdown of MAP1B significantly impaired cellular growth, migration, and invasive capacity(**Figures 6A–C**).

Phosphorylated MAP1B is crucial for maintaining proper microtubule dynamics and plays a pivotal role in cytoskeletal rearrangements that accompany neuronal differentiation and neurite outgrowth (36). However, in tumor cells, phosphorylated MAP1B has been shown to destabilize microtubules, thereby promoting tumor cell proliferation (37, 38). By analyzing the Wiki-pathways in both the shCDK1 and shCtrl groups, we found that the differential phosphoproteins were significantly enriched in signaling pathways related to microtubule cytoskeleton regulation(**Figure 6D**). Based on these observations, we speculate that CDK1 may promote the phosphorylation of MAP1B, leading to microtubule destabilization and enhanced proliferation of GBM cells. To further investigate the effects of shCDK1 on microtubule stability, we detected the immunofluorescence of acetylated alpha tubulin at U251 cells. The level of acetylated alpha tubulin, an indicator of stabilized microtubules, was significantly increased in shCDK1 cells (**Figure 6E**). This result indicates CDK1 inhibition promotes microtubule stability and a consequent suppression of cell proliferation.

In summary, our results indicate that interfering with CDK1 can indeed reduce MAP1B phosphorylation, thereby promoting microtubule stability and inhibiting cell proliferation.

## 4 Discussion

Glioblastoma (GBM) remains one of the most lethal malignancies of the central nervous system, characterized by aggressive proliferation, extensive invasion into surrounding brain tissue, and marked therapeutic resistance. In this study, we systematically investigated the oncogenic role of CDK1 in GBM through functional experiments and global phosphoproteomic profiling. Our findings revealed several critical insights: (i) CDK1 is significantly overexpressed in GBM tissues; (ii) knockdown of CDK1 markedly inhibits proliferation of GBM cells; (iii) CDK1 regulates a broad phosphoproteome encompassing 2,836 phosphorylation sites on key proteins involved in cell cycle regulation, DNA replication, and DNA damage repair; (iv) CDK1-mediated phosphorylation of MAP1B destabilizes microtubules and enhances tumor progression; and (v) hyperphosphorylation of MAP1B strongly correlates with poor prognosis in GBM patients. Collectively, these results establish CDK1 as a central oncogenic driver in GBM and identify MAP1B phosphorylation as a novel prognostic biomarker and potential therapeutic target.

### 4.1 CDK1 as a master regulator of GBM progression

CDK1, in complex with cyclin B, is the only essential cyclin-dependent kinase required for cell cycle progression in mammalian cells. Its canonical role in driving the G2/M transition is well established (39–44). Our data confirm that CDK1 is markedly overexpressed in GBM, consistent with previous reports demonstrating its upregulation across various solid tumors (45, 46). The strong reduction in proliferation and invasion following CDK1 knockdown highlights its indispensable role in GBM biology. Importantly, our phosphoproteomic analysis extends these observations by providing a global view of CDK1-regulated phosphorylation events. The enrichment of pathways related to DNA replication, DNA damage repair, and cell cycle progression underscores the multifaceted role of CDK1 beyond mitotic entry.

Notably, phosphorylation changes were enriched in transcription factors such as RB1 and TP53. RB1, a canonical regulator of G1/S transition, is classically phosphorylated by CDK4/6, but we observed reduced phosphorylation following CDK1 knockdown, suggesting additional layers of regulation. Similarly, TP53, a tumor suppressor frequently altered in GBM, showed decreased phosphorylation at Ser6 and Ser315, both reported CDK1 substrates (47–53). These findings indicate that CDK1 integrates signals across cell cycle checkpoints and tumor suppressor networks, thereby promoting oncogenic plasticity in GBM cells.

### 4.2 Novel role of CDK1 in regulating MAP1B phosphorylation

One of the most striking findings of this study is the identification of MAP1B as a CDK1-regulated substrate with strong prognostic implications. MAP1B is primarily known for its role in neuronal development, axonal growth, and microtubule dynamics (54, 55).

While MAP1B has been implicated in certain cancers, its phosphorylation-dependent role in GBM has not been systematically studied (56). Our data demonstrate that CDK1-mediated hyperphosphorylation of MAP1B at multiple sites (Ser832, Ser1260, Ser1899, Ser1939, Ser2209, Ser2271) is associated with microtubule destabilization and enhanced tumor proliferation. Functional assays confirmed that MAP1B knockdown significantly impaired GBM cell growth, migration, and invasion, validating its oncogenic role.

Mechanistically, phosphorylated MAP1B is known to increase the dynamics of microtubules by destabilizing the lattice and promoting disassembly (37, 57). In neurons, this is essential for axonal remodeling; in tumor cells, however, such destabilization provides a growth advantage by facilitating rapid cytoskeletal rearrangements, mitotic spindle formation, and invasive motility (58). Our findings thus reveal a previously unappreciated mechanism by which CDK1 promotes GBM progression—through modulation of cytoskeletal dynamics via MAP1B phosphorylation. This observation not only expands the spectrum of CDK1 substrates but also uncovers a novel signaling axis that links cell cycle regulation with microtubule remodeling in cancer.

### 4.3 Clinical implications: CDK1 and MAP1B as therapeutic targets

The clinical relevance of CDK1 and MAP1B is underscored by our survival analysis using CPTAC datasets, which demonstrated that hyperphosphorylated MAP1B correlates with significantly worse prognosis in GBM patients. These results suggest that MAP1B phosphorylation could serve as a prognostic biomarker for patient stratification.

From a therapeutic perspective, CDK1 has long been considered a challenging target due to its essential role in normal cell cycle regulation. However, accumulating evidence indicates that cancer cells, particularly those with deregulated checkpoints, are more dependent on CDK1 activity than normal cells—a phenomenon known as “oncogene addiction” (59). Our findings reinforce this concept and suggest that pharmacological inhibition of CDK1 could selectively impair GBM growth. Indeed, several CDK1 inhibitors are in preclinical or early clinical development (7). The discovery of MAP1B as a downstream effector of CDK1 also opens new therapeutic opportunities. Microtubule-targeting agents such as taxanes and vinca alkaloids are widely used in oncology, but their efficacy in GBM has been limited by blood–brain barrier penetration and systemic toxicity (60). Combining CDK1 inhibitors with microtubule-stabilizing agents may synergistically suppress GBM progression by simultaneously reducing MAP1B phosphorylation and stabilizing the microtubule network. Such combination strategies warrant further preclinical evaluation.

### 4.4 Limitations of the study

Despite its strengths, this study has several limitations. First, our functional validation was primarily conducted in the U251 GBM cell line, which may not fully capture the heterogeneity of GBM. Future studies using patient-derived xenografts or organoid models will be essential to confirm the generalizability of our findings. Second, the MAP1B site mutation experiment was not conducted, MAP1B phosphorylation sites should be experimentally perturbed (serine to alanine/glutamate mutagenesis) to establish their causal role in modulating microtubule dynamics in U251 cells. Additionally, *in vivo* studies will be crucial to evaluate the efficacy of CDK1-MAP1B targeting in GBM models.

### 4.5 Future directions

Moving forward, several avenues of research emerge from our findings. Elucidating the structural basis of CDK1 and MAP1B interaction could reveal specific phosphorylation motifs amenable to targeted inhibition. Given the complexity of microtubule regulation, it will be important to explore whether MAP1B phosphorylation cooperates with other cytoskeletal regulators in driving GBM invasion. Additionally, integrated proteogenomic analyses across larger patient cohorts may uncover subgroups of GBM patients most likely to benefit from CDK1-targeted therapies. Finally, preclinical studies testing CDK1 inhibitors, alone or in combination with microtubule-stabilizing drugs, should be pursued to translate our findings into therapeutic strategies.

## 5 Conclusion

In summary, this study highlights the pivotal role of CDK1 in GBM pathogenesis through comprehensive phosphoproteomic profiling. We demonstrate that CDK1 overexpression promotes GBM proliferation and invasion, and identify MAP1B hyperphosphorylation as a novel mechanism linking CDK1 activity to microtubule destabilization and tumor progression. MAP1B phosphorylation not only serves as a potential prognostic biomarker but also represents a new targetable vulnerability in GBM. These findings provide a strong rationale for the development of CDK1-targeted therapies, particularly in rational combinations with microtubule-stabilizing agents, to improve outcomes for patients with this devastating disease.

## Data Availability

The phosphoproteomics raw data presented in the study are deposited at integrated proteome resources (iProX) under accession IPX0014903001 or through the link (https://www.iprox.cn/page/DSV021.html;?url=1766927310562FwtR with the password ioJ8).
