# Supplementary figures and images for: CDK1-driven phosphorylation networks promote glioblastoma progression via MAP1B-mediated microtubule destabilization
Source: Front Oncol. 2026 Jan 14;15:1646698. doi: 10.3389/fonc.2025.1646698 (PMC12846947; doi:10.3389/fonc.2025.1646698)

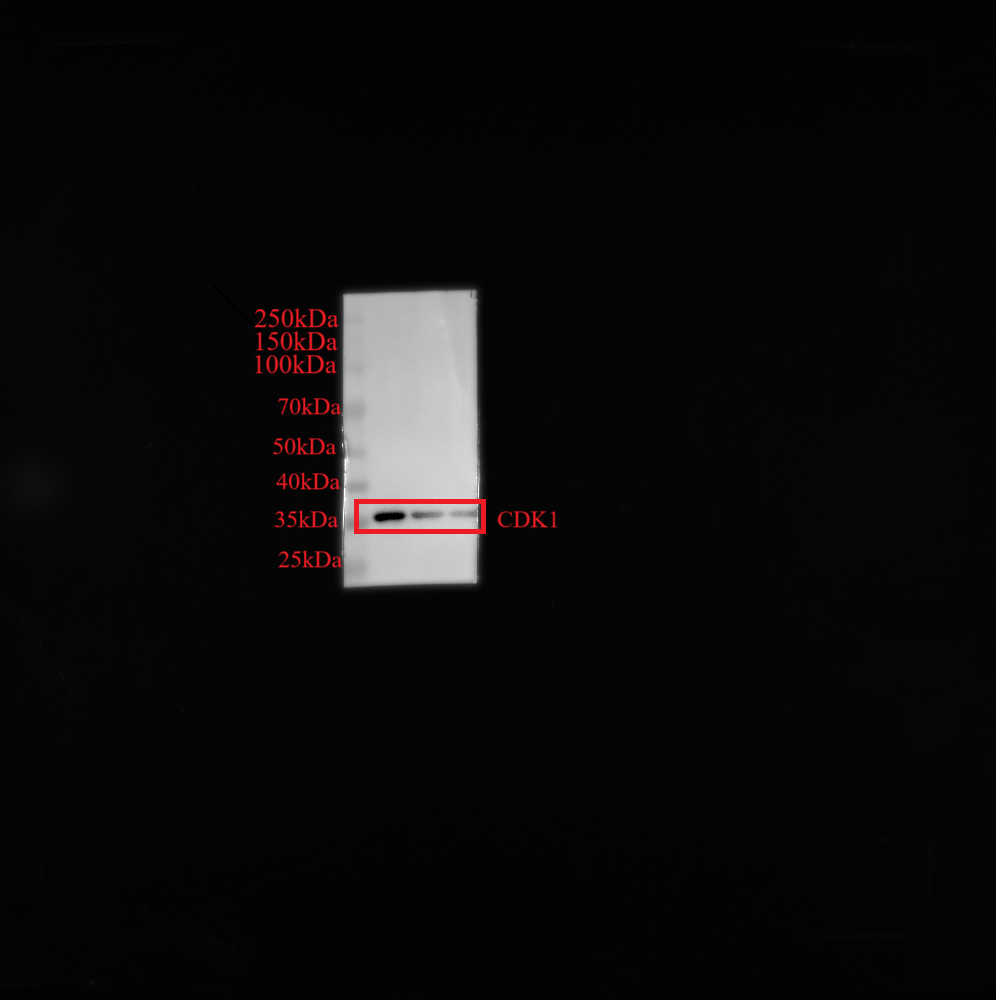

Supplement: Supplementary file 1 [file DataSheet1.zip › Fig6E CDK1.tif.tif]

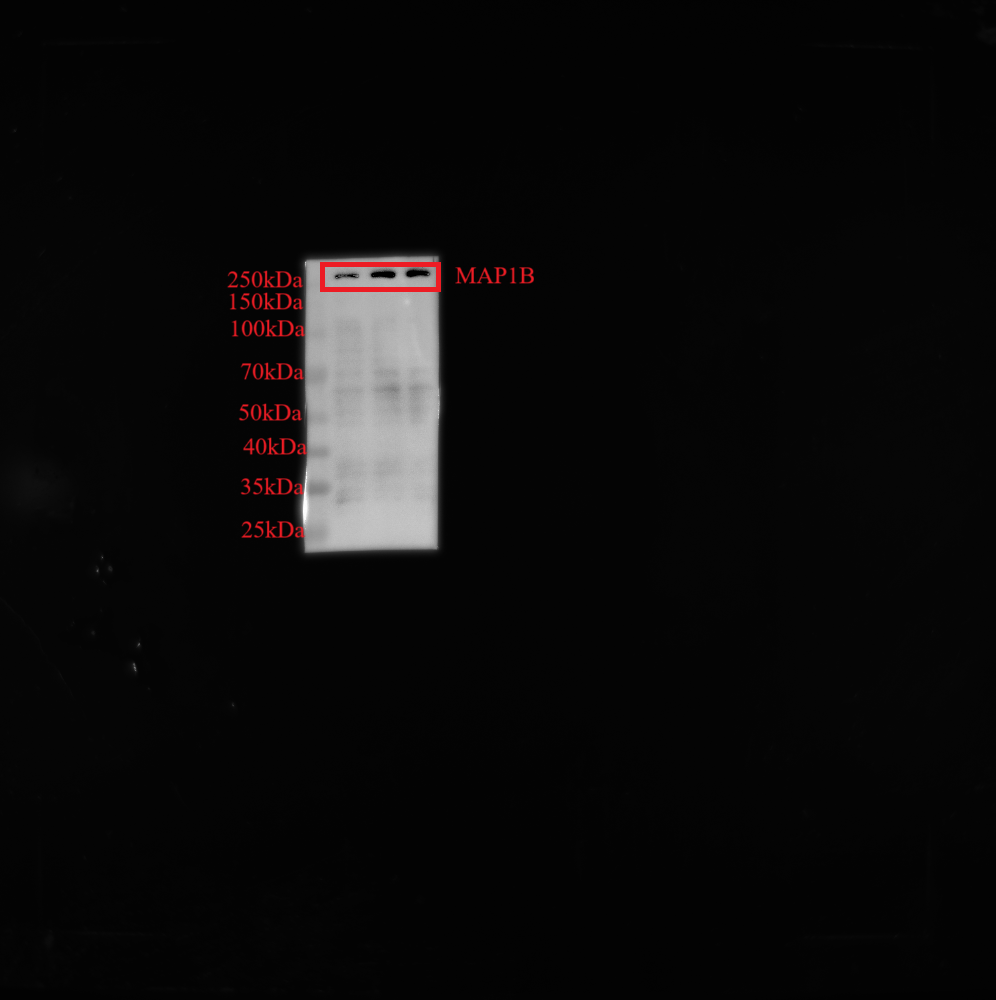

Supplement: Supplementary file 1 [file DataSheet1.zip › Fig6E MAP1B.tif.tif]

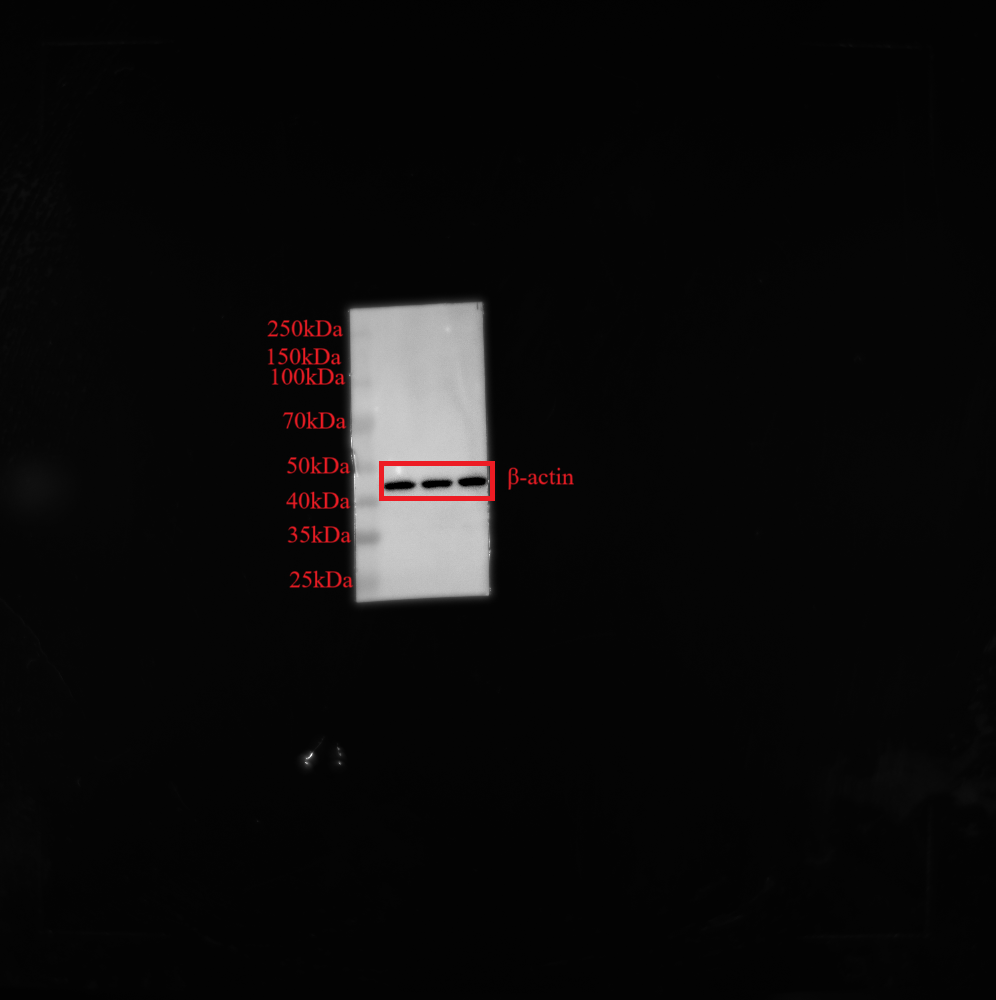

Supplement: Supplementary file 1 [file DataSheet1.zip › Fig6E β-actin.tif.tif]

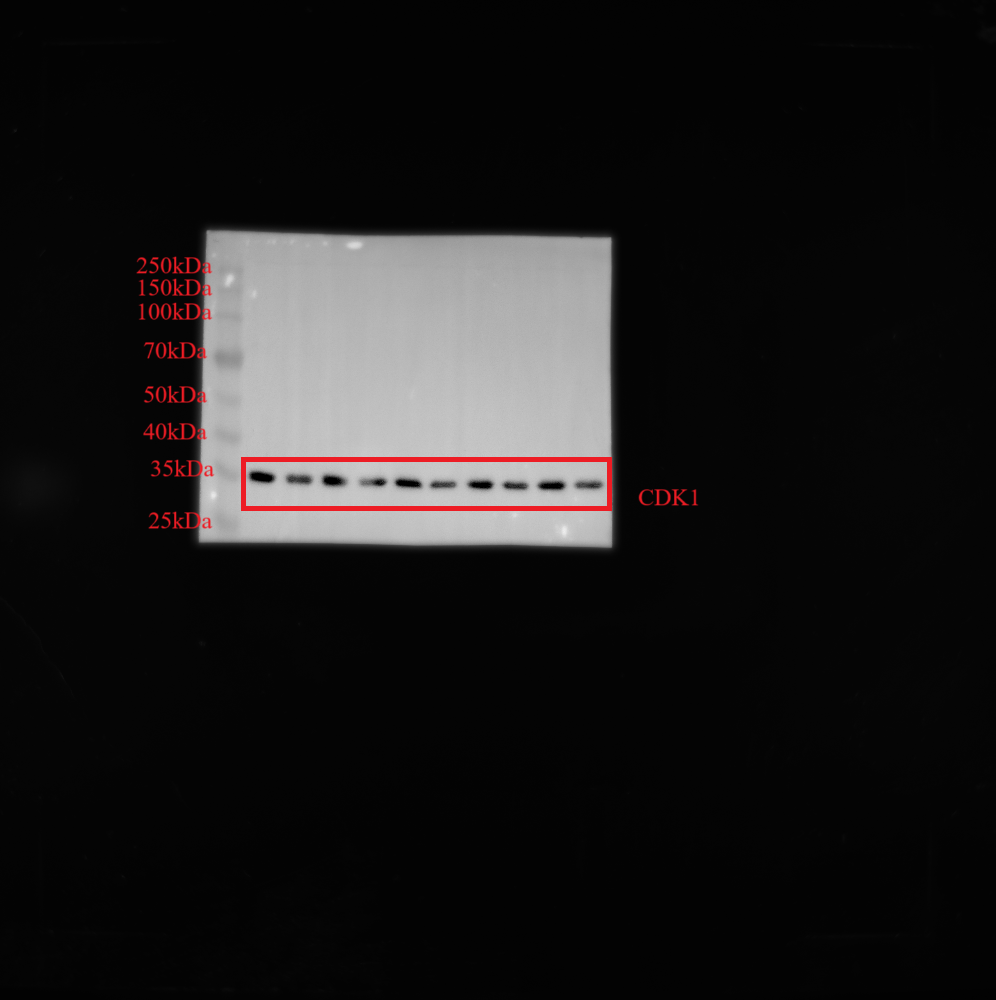

Supplement: Supplementary file 1 [file DataSheet1.zip › Fig1B CDK1.tif.tif]

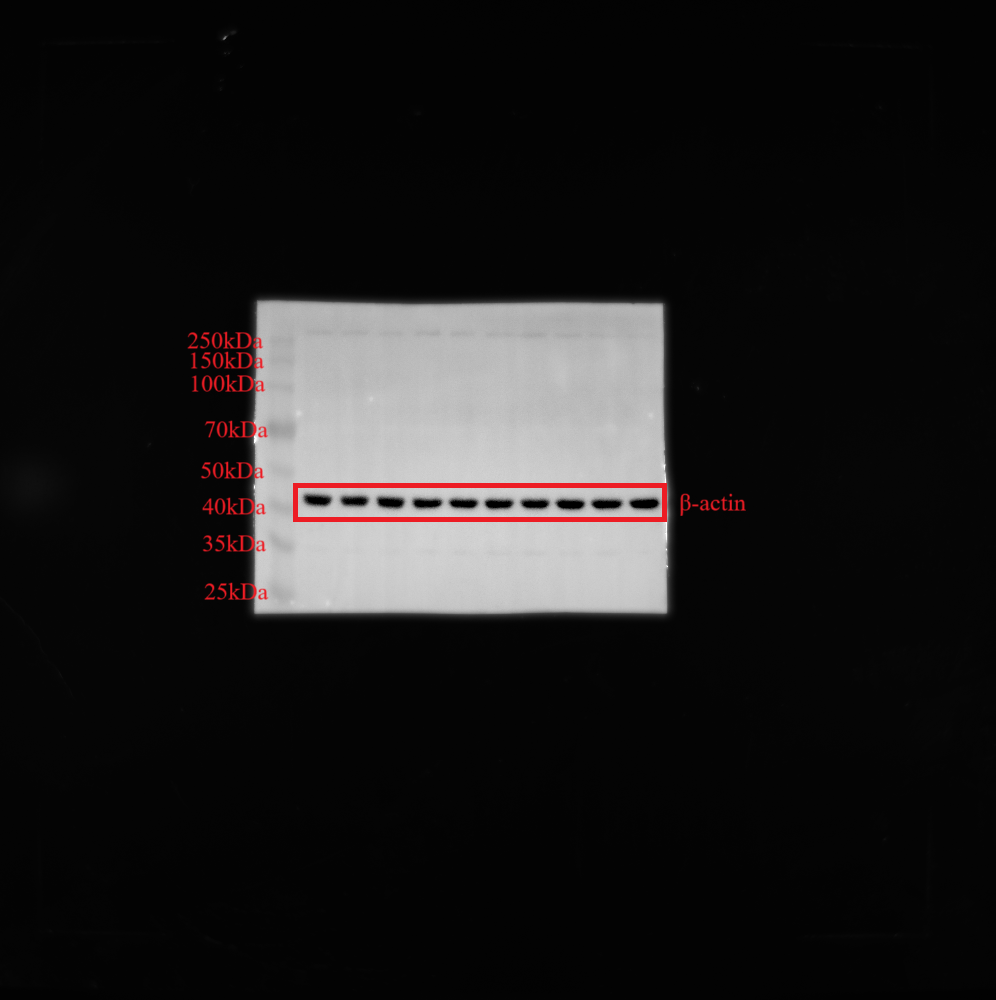

Supplement: Supplementary file 1 [file DataSheet1.zip › Fig1B β-actin.tif.tif]

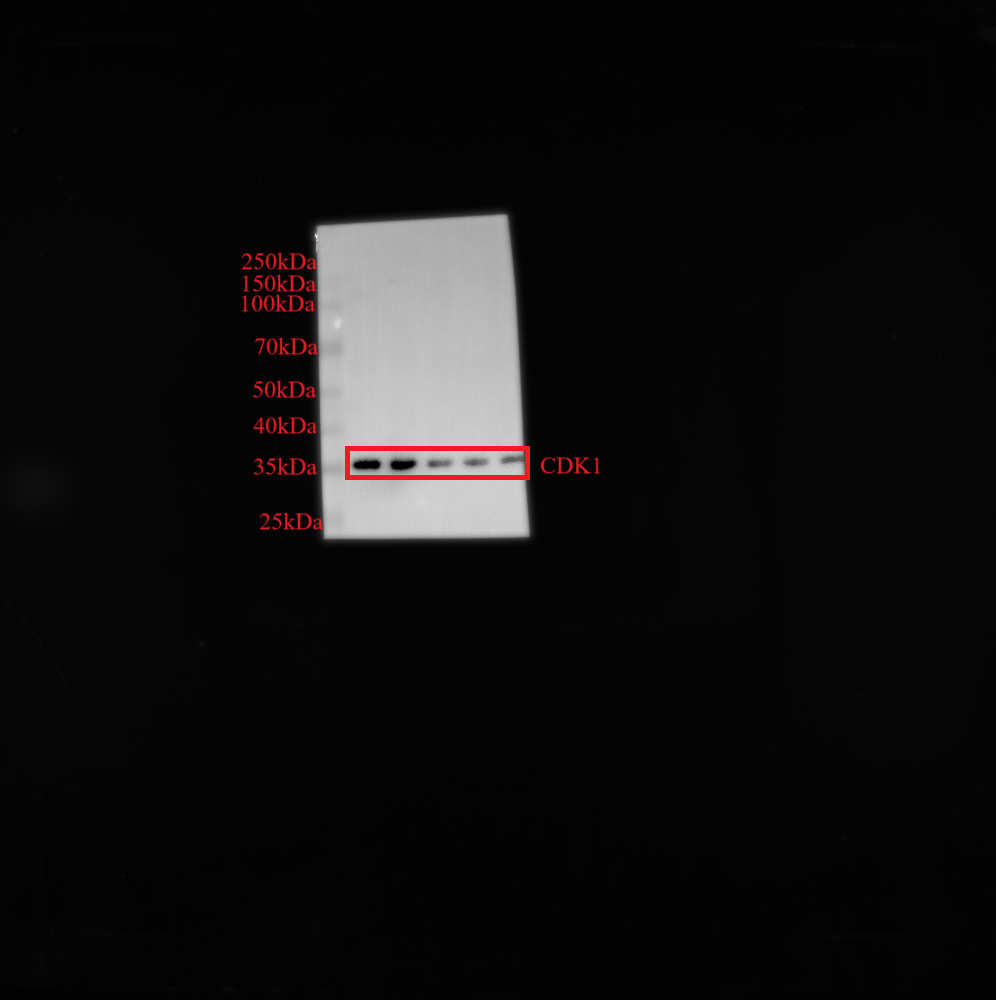

Supplement: Supplementary file 1 [file DataSheet1.zip › Fig1D CDK1.tif.tif]

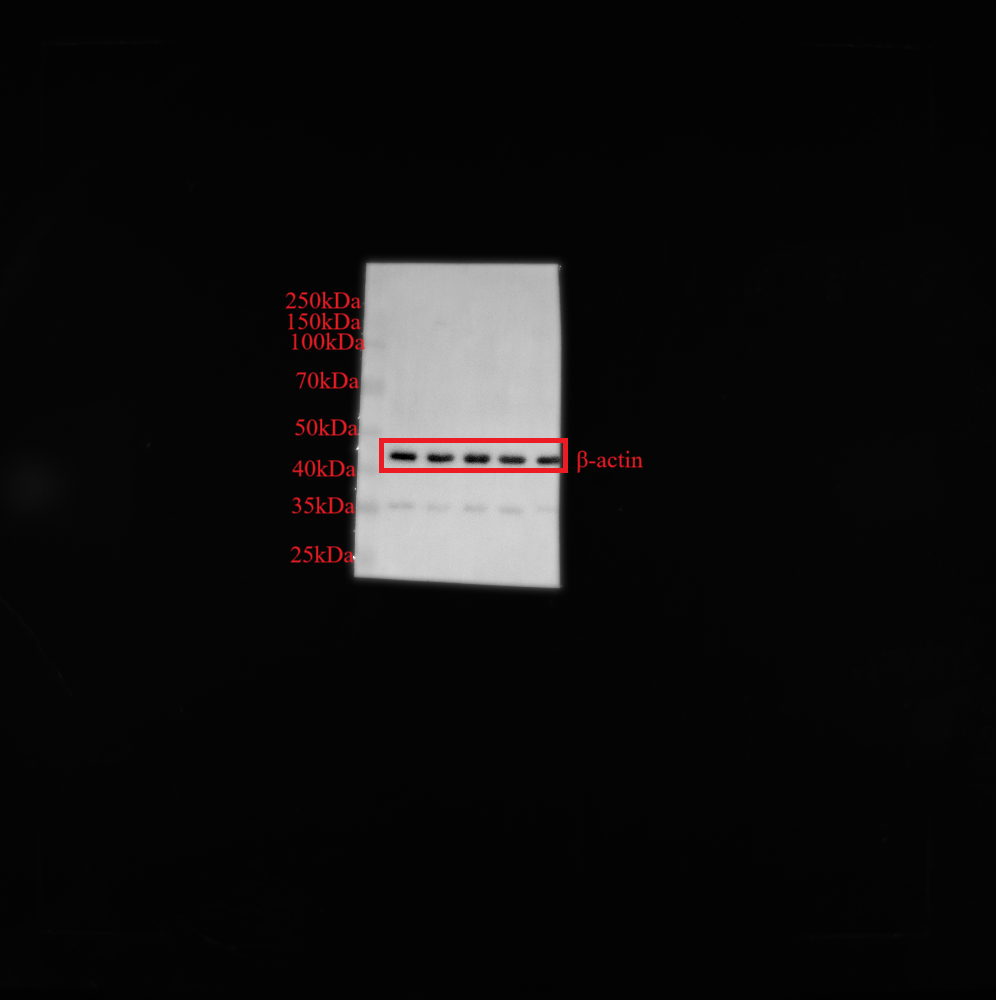

Supplement: Supplementary file 1 [file DataSheet1.zip › Fig1D β-actin.tif.tif]
